# Splenic Peliosis as a Rare Cause of Spontaneous Splenic Rupture: A Case Report

**DOI:** 10.7759/cureus.55839

**Published:** 2024-03-09

**Authors:** Amal Bakhsh, Hussain Ghandourah, Khatoon Alakrawi, Eman Alsahafi, Rana Saklou

**Affiliations:** 1 Department of Diagnostic Radiology, King Fahad General Hospital, Jeddah, SAU; 2 Department of General Surgery, King Fahad General Hospital, Jeddah, SAU

**Keywords:** blood cystic cavities, reticuloendothelial system, isolated peliosis, atraumatic splenic rupture, splenic peliosis

## Abstract

Atraumatic splenic rupture is a serious intraabdominal emergency that requires emergent intervention. This can be due to a number of causes. In this case report, we introduce a rare cause of atraumatic splenic rupture, which is an otherwise benign asymptomatic disease that only manifests clinically upon rupture, namely splenic peliosis. There is limited existing knowledge concerning the disease's etiology and diagnosis; however, this study presents the possible etiological explanations, associated risk factors, and possible radiologic diagnostic modalities.

## Introduction

The spleen is a commonly injured intraperitoneal organ within a trauma setting where quick radiological diagnosis is key in reducing mortality rates. Free fluid from intraperitoneal ruptures can be demonstrated with an examination called focused assessment with sonography for trauma (FAST). Yet, due to limitations posed by the US, multiphasic computed tomography is the modality of choice in assessing splenic ruptures [1].

Splenic ruptures can occur in a non-traumatic setting as well, though they occur rarely, with incidence rates of 0.1-0.5 % [2]. Causes of atraumatic splenic rupture (ASR) are vast, but the most common causes include infections such as Malaria, Cytomegalovirus, and Epstein-Barr virus, coagulopathies, such as anticoagulant use, idiopathic thrombocytopenic purpura, and platelet deficiencies or neoplasms such as leukemias, lymphomas and metastases [3]. Our case discusses one of the rarer causes of ASR, namely splenic peliosis.

Splenic peliosis is an exceptionally rare benign disorder of unknown etiology occurring in organs associated with the reticuloendothelial system, such as the liver, spleen, bone marrow, and lymph nodes [4]. It most commonly occurs in the liver and very rarely in the spleen. It is characterized by the presence of multiple cystic blood-filled cavities that are susceptible to rupture, leading to life-threatening hemorrhage and presenting with an acute abdomen. Without rupture, the disease is asymptomatic and only discovered incidentally. 

Splenic peliosis is reported to be associated with conditions such as tuberculosis, hematologic malignancies, immunodeficiencies, intravenous drug abuse, and chronic alcoholism in combination with either contraceptive or steroid use [5]. We present a case of a patient with emergent ASR at our hospital with no comorbidities or known underlying contributing pathology, later to be diagnosed with splenic peliosis.

## Case presentation

The patient is a 30-year-old Egyptian male with no previous surgical history or underlying medical conditions. Social history is only significant for tobacco smoking. This patient presented to the emergency department at King Fahad General Hospital in Jeddah, Saudi Arabia, with the chief complaint of abdominal pain. He reported that his pain started on the same day of his presentation, described as a sudden and continuous sharp pain localized to the left upper quadrant of the abdomen, after which it shifted to the lower abdomen.

At presentation, the patient denied any associated symptoms, such as vomiting, fever, chills, diarrhea, constipation, or other gastrointestinal symptoms. Furthermore, a history of abdominal trauma had been denied. On initial assessment, his vitals showed a heart rate of 79 beats per minute, a blood pressure of 110/65 mmHg, a respiratory rate of 13 breaths per minute, a temperature of 36.8° C, and oxygen saturation of 98% in room air. Abdominal examination revealed a non-distended abdomen with tenderness localized to the left upper quadrant, without rebound tenderness or guarding.

Baseline laboratory investigation showed hemoglobin of 12.9 g/dl (13-17), white blood count of 11.5x10^9/L (4-10), platelets of 136x10^9/L (150-410), coagulation and serum electrolytes were within normal range.

Enhanced computed tomography of the abdomen in the portal venous phase was performed and showed an enlarged spleen measuring up to 20 cm with an irregular small hypodensity in the posterior inferior aspect, measuring 2.5x2 cm associated with significant peri splenic high-density fluid ( >50 HU) and areas of contrast enhancement, likely representing contrast extravasation (active bleeding).

**Figure 1:**
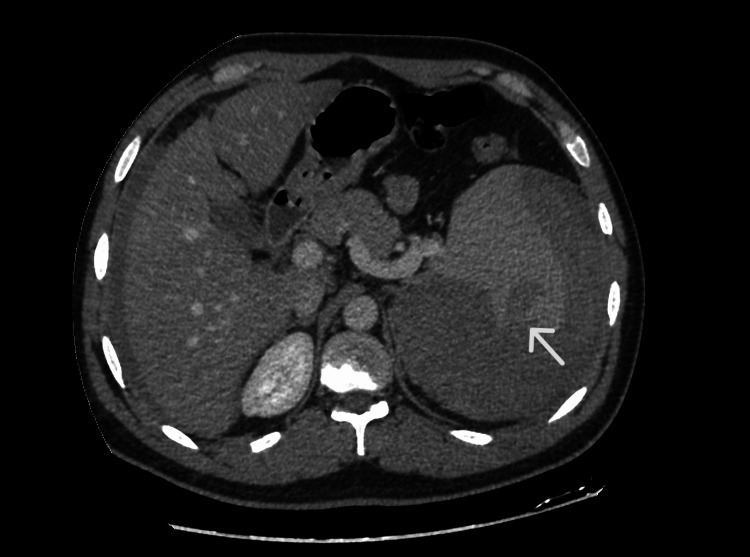
Enhanced CT shows a hypodense lesion through the posterior inferior portion of the spleen with peri-splenic hematoma and abdominal free fluid

**Figure 2:**
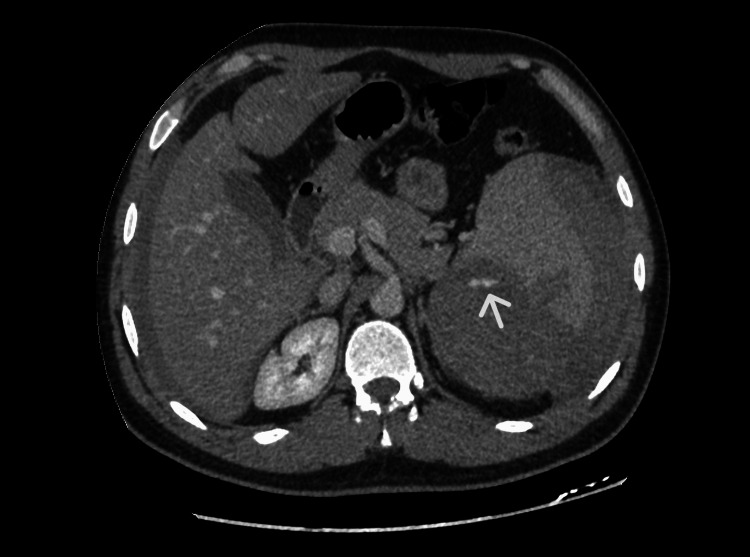
Enhanced CT shows areas of contrast enhancement seen more posterior and inferior to the spleen and adjacent to the splenic hypodensity

Bilateral moderate dense fluid was noted in the perihepatic and peri splenic space extending to the paracolic gutters and pelvis, in keeping with hemoperitoneum (Figures 1 and 2).

Upon re-evaluation, the patient became pale, and vital signs revealed a heart rate of 120 beats per minute, blood pressure of 86/55 mmHg, respiratory rate of 17 breaths per minute, temperature of 37° C, and oxygen saturation of 98% in room air.

The diagnosis of spontaneous splenic rupture was made, and therefore, the patient was taken to the operating theatre for exploratory laparotomy and splenectomy. Intra-operative findings included a massive hemoperitoneum of around 2500cc, a friable large spleen that was hardly mobilized due to its consistency, and a splenule over the omentum. 

**Figure 3:**
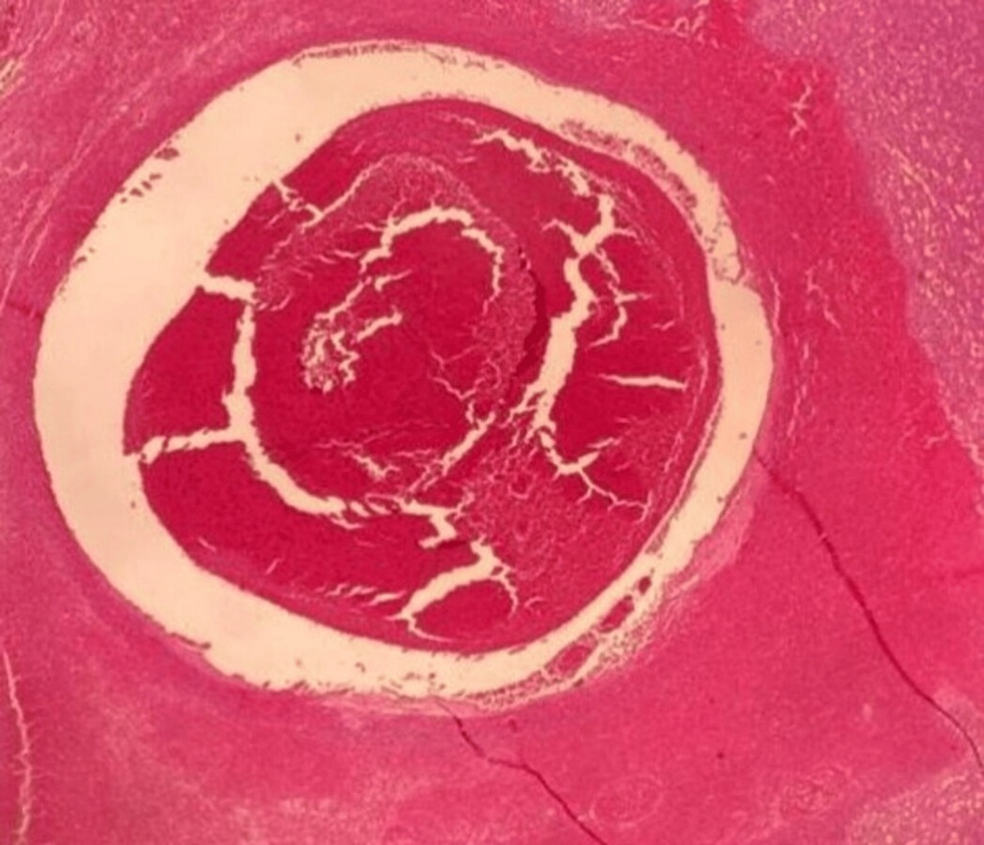
Histopathology shows areas of blood pooling rich in neutrophils

The spleen (weighing 567 g) was sent for histopathology evaluation, which showed focal areas of blood pooling rich in neutrophils, as seen in Figure 3, supporting the diagnosis of splenic peliosis.

The patient remained well after surgery and was discharged home on day 10 after surgery.

## Discussion

The term peliosis was first introduced in literature in 1916 by Schoenlank to describe diffuse circular bluish-reddish-blackish colored clotted liver lesions made of fine fibrin networks, introduced as peliosis hepatis [6]. However, the first description of splenic peliosis dates back to 1866 when Cohnheim described a sudden mortality case involving a 27-year-old patient in a psychiatric ward where an autopsy revealed hemoperitoneum due to a splenic rupture. The autopsy report further mentioned multiple cystic lesions with clotted blood within the spleen [7]. Splenic peliosis is incidental and asymptomatic, presenting symptoms only upon rupture with an approximate incidence rate of 1%, as seen in autopsy reports [8].

Splenic peliosis has been reported to be associated with human immunodeficiency virus (HIV), liver cirrhosis, tuberculosis, hematologic malignancies such as Hodgkin's lymphoma, myeloma and aplastic anemia, intravenous drug abuse, alcoholism, and use of certain medications such as anabolic steroids and oral contraceptive pills [9,10]. It was also found in patients with concurrent conditions such as diabetes mellitus, end-stage renal disease, and renal transplantation [11]. However, no associated conditions or risk factors for splenic peliosis have been identified in our patient.

A previous case report from 2016 revealed similar clinical features where the patient experienced left upper quadrant abdominal pain and was hemodynamically stable. Laboratory tests showed a hemoglobin of 9.8 g.dL. Initial ultrasound showed a 3x3 cm heterogenous lesion in the spleen along with free fluid. CT scan of the abdomen demonstrated a grade III laceration of the spleen with moderate hemoperitoneum. The patient then deteriorated during the next 12 hours and was planned for urgent exploratory laparotomy. There was about 500 cc of frank blood in the peritoneal cavity, and the spleen was found ruptured; therefore, a splenectomy was performed. The patient remained well and was discharged on the fifth day after surgery. Upon histopathological evaluation of the spleen, there were dilated spaces filled with hemorrhage and fibrin, which was consistent with splenic peliosis [4].

Since then, there have been a few publications discussing the disease's pathogenesis, yet it remains under debate. The pathogenesis of splenic peliosis is currently undetermined. However, several theories have been hypothesized since its discovery, one of which is that the origin of lesions is due to venous malformations arising due to abnormal intravascular pressure conditions within the spleen. These venous abnormalities could be due to an acquired cause or an embryogenic maldevelopment cause [8].

Possible differentials of splenic peliosis include hemangiomas and hairy cell leukemia. Hemangiomas consist of vascular channels with endothelial lining adjacently separated by fibrous septa. Meanwhile, in hairy cell leukemia, the endothelial lining is replaced by hairy cells instead. Other possible differentials include angiomatous lesions, such as lymphangioma, angiosarcoma, and bacillary angiomatosis. Splenic peliosis is differentiated from other cystic lesions by the presence of numerous scattered blood-filled cysts found in the red pulp with the predominant involvement of splenic parafollicular zones [12].

Splenic peliosis can be diagnosed microscopically by histopathology and autopsy or radiologically. Ultrasound shows ill-defined foci of varying hypoechoic or hyperechoic lesions, with larger lesions showing more pronounced posterior acoustic enhancement [13]. Pre-contrast computerized tomography (CT) presents the blood-filled cysts as hypoattenuating multiloculated lesions with well-defined septa. These lesions slowly and avidly enhance post-contrast in a centripetal pattern with subsequent loss of the visualized lobulations and septa. The lesions do not appear calcified, and extra-capsular extensions of these lesions are not seen unless they rupture, resulting in subcapsular hematoma and intraperitoneal rupture [13-14]. Magnetic resonance imaging (MRI) on the T2 sequence shows mixed signal intensities due to the cystic contents, primarily containing deoxyhemoglobin and methemoglobin [15]. The differentials based on radiologic appearance include hemangiomas, lymphangioma, and angiosarcoma.

Surgical management or possible prophylaxis of splenic peliosis is not well understood in existing literature. Upon successful surgical management of post-splenic rupture and an established diagnosis, the presence of peliosis can subsequently be identified in different organs to prevent further future complications and possibly identify associated conditions as well, such as possible malignancies. Possible lifestyle changes can be additionally advised, such as discontinuing oral contraceptive use or anabolic steroids in certain patient populations, as well as avoiding high-contact sports.

## Conclusions

Splenic peliosis is a rare and asymptomatic entity which can lead to sudden atraumatic splenic rupture. It is a silent incidental finding which usually only clinically manifests during rupture causing an acute symptomatic emergency presentation. Radiological detection is limited due to a possible number of other lesions with similar characteristics. The diagnosis is usually confirmed following splenectomy or autopsy.
